# Diagnostic value of partial exome sequencing in developmental disorders

**DOI:** 10.1371/journal.pone.0201041

**Published:** 2018-08-09

**Authors:** Laura Gieldon, Luisa Mackenroth, Anne-Karin Kahlert, Johannes R. Lemke, Joseph Porrmann, Jens Schallner, Maja von der Hagen, Susanne Markus, Sabine Weidensee, Barbara Novotna, Charlotte Soerensen, Barbara Klink, Johannes Wagner, Andreas Tzschach, Arne Jahn, Franziska Kuhlee, Karl Hackmann, Evelin Schrock, Nataliya Di Donato, Andreas Rump

**Affiliations:** 1 Institut für Klinische Genetik, Medizinische Fakultät Carl Gustav Carus, Dresden, Technische Universität Dresden, Germany; 2 Klinik für angeborene Herzfehler und Kinderkardiologie, Universitätsklinikum Schleswig-Holstein, Campus Kiel, Kiel, Germany; 3 Institut für Humangenetik, Universitätsklinikum Leipzig, Leipzig, Germany; 4 Abteilung Neuropädiatrie, Medizinische Fakultät Carl Gustav Carus, Technische Universität Dresden, Dresden, Germany; 5 MVZ Dr. Staber und Kollegen, Regensburg, Germany; 6 Mitteldeutscher Praxisverbund Humangenetik, Praxis Erfurt, Erfurt, Germany; King Faisal Specialist Hospital and Research Center, SAUDI ARABIA

## Abstract

Although intellectual disability is one of the major indications for genetic counselling, there are no homogenous diagnostic algorithms for molecular testing. While whole exome sequencing is increasingly applied, we questioned whether analyzing a partial exome, enriched for genes associated with Mendelian disorders, might be a valid alternative approach that yields similar detection rates but requires less sequencing capacities. Within this context 106 patients with different intellectual disability forms were analyzed for mutations in 4.813 genes after pre-exclusion of copy number variations by array-CGH. Subsequent variant interpretation was performed in accordance with the ACMG guidelines. By this, a molecular diagnosis was established in 34% of cases and candidate mutations were identified in additional 24% of patients. Detection rates of causative mutations were above 30%, regardless of further symptoms, except for patients with seizures (23%). We did not detect an advantage from partial exome sequencing for patients with severe intellectual disability (36%) as compared to those with mild intellectual disability (44%). Specific clinical diagnoses pre-existed for 20 patients. Of these, 5 could be confirmed and an additional 6 cases could be solved, but showed mutations in other genes than initially suspected. In conclusion partial exome sequencing solved >30% of intellectual disability cases, which is similar to published rates obtained by whole exome sequencing. The approach therefore proved to be a valid alternative to whole exome sequencing for molecular diagnostics in this cohort. The method proved equally suitable for both syndromic and non-syndromic intellectual disability forms of all severity grades.

## Introduction

The American Association on Intellectual and Developmental Disability defines intellectual disability (ID) as significant limitation both in intellectual functioning and in adaptive behavior. This encompasses limited conceptual, social and practical adaptive skills originating before the age of 18 years. In children younger than 5 years the term global developmental delay (DD) is used to indicate a significant delay in 2 or more developmental domains [1]. ID/DD with or without additional anomalies is the most frequent reason for genetic counselling [2].

The recent evaluation approach for children with DD recommended by the American Academy of Pediatrics in 2014 applies a so-called phenotype-first approach and suggests a chromosomal microarray as a first tier universal test. Whole exome sequencing (WES), as an emerging technology, is still discussed controversially [1]. However, WES is widely used in multiple diagnostic laboratories especially in the United States [3, 4].

The European Society of Human Genetics recommends the application of focused gene panels and the evaluation of test results deploying a next generation sequencing (NGS)-specific rating system [5]. According to this system, ideal diagnostic analyses (type A tests) should guarantee > 99% reliable variant calls within coding and flanking intronic regions, with all the gaps covered by another complementary sequencing technique [5]. High and universal coverage can best be reached by carefully designed multi-gene panels [6].

In this study we report our experiences with partial exome sequencing using Illumina’s TruSight-One (TS1) panel in a cohort of 106 undiagnosed patients with different degrees of ID/DD and/or multiple congenital anomalies (MCA) that were recruited by a single center.

## Materials and methods

### Subjects

We screened a cohort of 106 unrelated patients with a median age of 6.8 years, including 5 fetal samples from terminated pregnancies. Fetal samples were included either because of a brain malformation that was observed by ultrasound, MRI scan or autopsy or because there was suspicion of a clinical diagnosis known to cause ID/DD. The cohort included 47 (44.9%, CI 35.24% - 53.83%) female and 59 (55.7%, CI 46.17% to 64.76%) male index patients from unrelated families of predominantly German (northwestern European) ancestry. Consanguinity was reported in only 1 of the 106 families. Informed written consent was obtained from all patients/legal guardians prior to genetic testing in accordance with the German law (Genetic Diagnosis Act, GenDG). This study was approved by the ethics committee Leipzig (ethics statements 226/16-ek and 402/16-ek). The legal guardians of the individuals depicted in photographs in this manuscript have given written informed consent (as outlined in PLOS consent form) to publish these case details.

### Clinical data and prior testing

Clinical datasets including photographs were available from all the patients. We personally examined 94 of 106 patients (dysmorphologic evaluation was done by NDD and AT). Only patients without prior clinical diagnosis or with a tentative diagnosis requiring testing of multiple genes were included in the study. Prior to NGS all patients received conventional karyotyping and chromosomal microarray (Human CGH Microarray Kit 2x400k, design 21850, Agilent Santa Clara, CA), showing normal results. Additionally, all male patients (except fetal cases) were screened negative for Fragile X syndrome.

The most common clinical feature and referral reason for panel sequencing was ID/DD (98 of 106 patients, 92.5%, CI 85.61% - 96.33%). The remaining 8 patients presented with a complex MCA syndrome.

ID/DD was severe in 39.6% (n = 42 / 106, CI 30.82% - 49.15%), moderate in 35.8% (n = 38 / 106, CI 27.35% - 45.34%) and mild in 17.0% (n = 18 / 106, CI 10.93%– 25.34%) of patients. Severity was assessed by a clinical geneticist in accordance with the Diagnostic and Statistical Manual of Mental Disorders (DSM –5), grouping together the DSM-5 categories “severe” and “profound” [7]. 91.5% (n = 43 / 47, CI 79.54%– 97.17%) of all females and 93.2% (n = 55 / 59, CI 83.36% - 97.80%) of all males were classified as syndromic, defined by the presence of at least one additional symptom besides ID/DD, such as minor facial anomalies (67.0%, n = 71 / 106, CI 57.55% - 75.22%), major anomalies (67.9%, n = 72 / 106, 58.52% - 76.07%) or seizures (29.2%, n = 31 / 106, CI 21.40% - 38.54%). Additional clinical features and proposed clinical tentative diagnoses are summarized in S1 Table.

### Partial exome sequencing

The coding exons of 4.813 OMIM listed genes, most of them associated with known clinical phenotypes, were enriched from 50 ng of blood-derived genomic DNA using the TruSight-One sequencing panel (TS1, Illumina, San Diego, CA) according to the manufacturer’s instructions. 150 nt paired-end sequencing was performed with a median target coverage of 80-fold either on an Illumina MiSeq or NextSeq sequencer. Reads were aligned to the reference genome (GRCh37/hg19) and the variant calling was performed using the CLC Biomedical Genomics Workbench (Qiagen, Hilden, Germany) as described previously [8]. Variants of interest were validated by Sanger sequencing.

In total we performed 265 analyses including 27 analyses of single patients, 78 trio analyses and 1 quartet (parents and 2 affected children). Single analyses were performed in cases where both or one of the parents was unavailable at the time of analysis and in cases with a specific preliminary diagnosis.

For single analyses the inheritance status was determined by Sanger sequencing of the parents, if available. On average 1.4 variants (ranging from 0–5 variants) were segregated per single TS1 analysis. In 6 of the single cases either only the mother or none of the parents was available for segregation analysis.

### Variant classification

Variant classification was performed according to ACMG criteria [9] by a team of clinicians and molecular geneticists (LG, LM, AKK, AR, NDD). In selected cases (S1 Table: Patient #3, Patient #4, Patient #31) additional metabolic tests were initiated to assess the potential effect on protein function. Variants were categorized as causative, incidental finding, variant of unknown significance (VUS) or variant in a gene of unknown significance (GUS).

Only pathogenic or likely pathogenic mutations in known Mendelian disease genes associated with the phenotype observed in the patient where categorized as causative. For diseases with an autosomal recessive inheritance pattern mutations were classed as causative if at least one of the variants was likely pathogenic or pathogenic according to ACMG criteria and a second variant in the same gene, proven to be on the other allele, was classified at least as VUS.

Mutations were categorized as incidental findings if the mutation did not explain the patients’ phenotype but was classified as either likely pathogenic or pathogenic by the use of ACMG criteria and affected a gene in which mutations are known to cause a well-defined phenotype.

Genes were classified as GUS if there were no reports of specific phenotypes caused by mutations in this gene or if the associated disease did not match the patient’s phenotype.

### Statistical analysis

95% confidence intervals (CI) were calculated for all diagnostic rates and differences between detection rates in patient groups (cohort vs. subgroups) were assessed by Fisher's Exact Test for count data. Statistical analysis was performed with Prism 7 for Mac. Statistical significance was defined as p < 0.05.

## Results

### General diagnostic yield

Mendeliome panel sequencing identified disease causing variants in 36 out of 106 patients (34.0%, CI 25.04% - 43.80%). This includes 20 patients with a specific prior clinical diagnosis, of which 5 could be confirmed on a molecular level. In another 6 of these patients a molecular diagnosis other than clinically suspected was found. These unexpected molecular findings are discussed below with the clinical reports summarized in S1 Clinical Data. In all, 11 out of 20 cases (55%, CI 34.19% - 74.19%) with a specific prior clinical diagnosis could be solved. The molecular cause was further identified in 26 of the remaining 86 patients with no prior specific clinical diagnosis (28.7%, CI 21.51%– 40.65%). These mutations cause 26 different clinical entities, of which 11 have earlier been described as syndromes that can clinically be recognized. An overview of the clinical information is given in Table 1 (detailed information to prior clinical diagnoses in S2 Table).

**Table 1:** Summary of clinical information and mutation detection rates for subgroups of the cohort.

	Cohort	Solved cases	Detection rate (p-values as compared to detection rate of whole cohort)
**Whole cohort**	**106 index patients**	**36 index patients**	n = 36 / 106 **(34.0%)** (CI 25.04% - 43.80%)
Average age	6.8 years	6.0 years	
Female	n = 47 / 106 (44.3%) (CI 35.24% - 53.83%)	n = 19 / 36 (52.7%) (CI 37.00% - 68.02%)	n = 19 / 47 (40.4%) (CI 27.62%– 54.68%, p = 0.4942)
Male	n = 59 / 106 (55.7%) (CI 46.17%– 64.76%)	n = 17 / 36 (47.2%) (31.98% - 63.00%)	n = 17 / 59 (28.8%) (CI 18.77%– 41.45%, p = 0.6023)
**ID**	**n = 98 / 106 (92.5%)** CI (85.61% - 96.33%)	**n = 33 / 36 (91.7%)** (CI 77.43% - 97.87%)	**n = 33 / 89 (33.7%)** (CI 25.07% - 43.51%, p >> 0.99)
Mild ID	n = 18 / 106 (17.0%) (CI 10.93% - 25.34%)	n = 8 / 36 (22.2%) (CI 11.47%– 38.33%)	n = 8 /18 (44.4%) (CI 24.54%– 66.30%, p = 0.4658)
Moderate ID	n = 38 /106 (35.8%) (CI 27.35% - 45.34%)	n = 10 / 36 (27.8%) (CI 15.70% - 44.14%)	n = 10 / 38 (26.3%) (CI 14.81%– 42.17%, p = 0.4208)
Severe ID	n = 42 / 106 (39.6%) (CI 30.82% - 49.15%)	n = 15 / 36 (41.7%) (CI 27.12% - 57.82%)	n = 15 / 42 (35.7%) (CI 22.94%– 50.88%, p = 0.8461)
**Additional symptoms**	** **	** **	** **
Facial anomaly	n = 71 / 106 (67.0%) (CI 57.55% - 75.22%)	n = 26 / 36 (72.2%) (CI 55.86% - 84.30%)	26 / 71 (36.6%) (CI 26,35%– 48.26%, p = 0.6796)
Major anomaly	n = 72 / 106 (67.9%) (CI 58.52%)– 76.07%)	n = 23 / 36 (63.9%) (CI 47.52% - 77.58%)	n = 23 / 72 (31.9%) (CI 22.29%– 43.43%, p = 0.685)
Brain malformation	n = 34 / 106 (32,1%) (CI 23.93%– 41.48%)	n = 12 / 36 (33.3%) (CI 20.14%– 49.74%)	n = 12 / 34 (35.3%) (CI 21.42%– 52.15%, p >> 0.99)
Abnormal body measurements	n = 67 / 106 (63.2%) (CI 53.70%– 71.79%)	n = 21 / 36 (58.3%) (CI 42.18% - 72.88%)	n = 21 / 67 (31.3%) (CI 21.46%– 43.25%, p = 0.6918)
Seizures	n = 31 / 106 (29.2%) (CI 21.40% - 38.50%)	n = 7 / 36 (19.4%) (CI 9.45–35.33%)	n = 7 / 31 (22.5%) (CI 11.11%– 40.10%) p = 0.2841
Neurologic features other than seizures	n = 20 / 106 (18.9%) (CI 12.48% - 27.43%)	n = 7 / 36 (19.4%) (CI 9.45–35.33%)	n = 7 / 20 (35.0%) (CI 15.39%– 59.22%) p >> 0.99)
Heart defects	n = 18 / 106 (17.0%) (CI 10.93%– 25.34%)	n = 7 / 36 (19.4%) (CI 9.45–35.33%)	n = 7 / 18 (38.9%) (CI 20.23%– 61.46%) p = 0.8011
Syndromic	n = 98 / 106 (92.5%) (CI 85.61% - 96.33%)	n = 34 / 36 (94.4%) (CI 80.91% - 99.41%)	n = 34 / 98 (34.7%) (CI 25.99%– 44.55% p >> 0.99)

### Diagnostic yield by phenotype

We found no difference between the diagnostic yields in different subgroups separated by sex, severity of ID/DD, occurrence of facial anomalies, major anomalies, brain malformations, abnormal body measurements, heart defects or neurological features others than seizures.

We did, however, observe, by trend, a lower mutation detection rate in the subgroup of patients who presented with seizures (22.5%, n = 7 / 31, CI 11.11%– 40.10%, p = 0.2841) than in the whole patient cohort. Moreover, the molecular cause could be clarified in only 2 out of 7 patients with early onset epileptic encephalopathy (28.6%, CI 7.56% - 64.76%, p >> 0.99).

We further noted that the correct clinical diagnosis was made in only 5 out of 36 solved cases (13.9%, CI 5.61% - 29.13%) prior to genetic testing. Moreover, in 6 patients the initial clinical diagnosis had to be revised according to the sequencing results (S2 Table). Here we recapitulate 3 cases that were considered to be of special interest.

**Fig 1:**
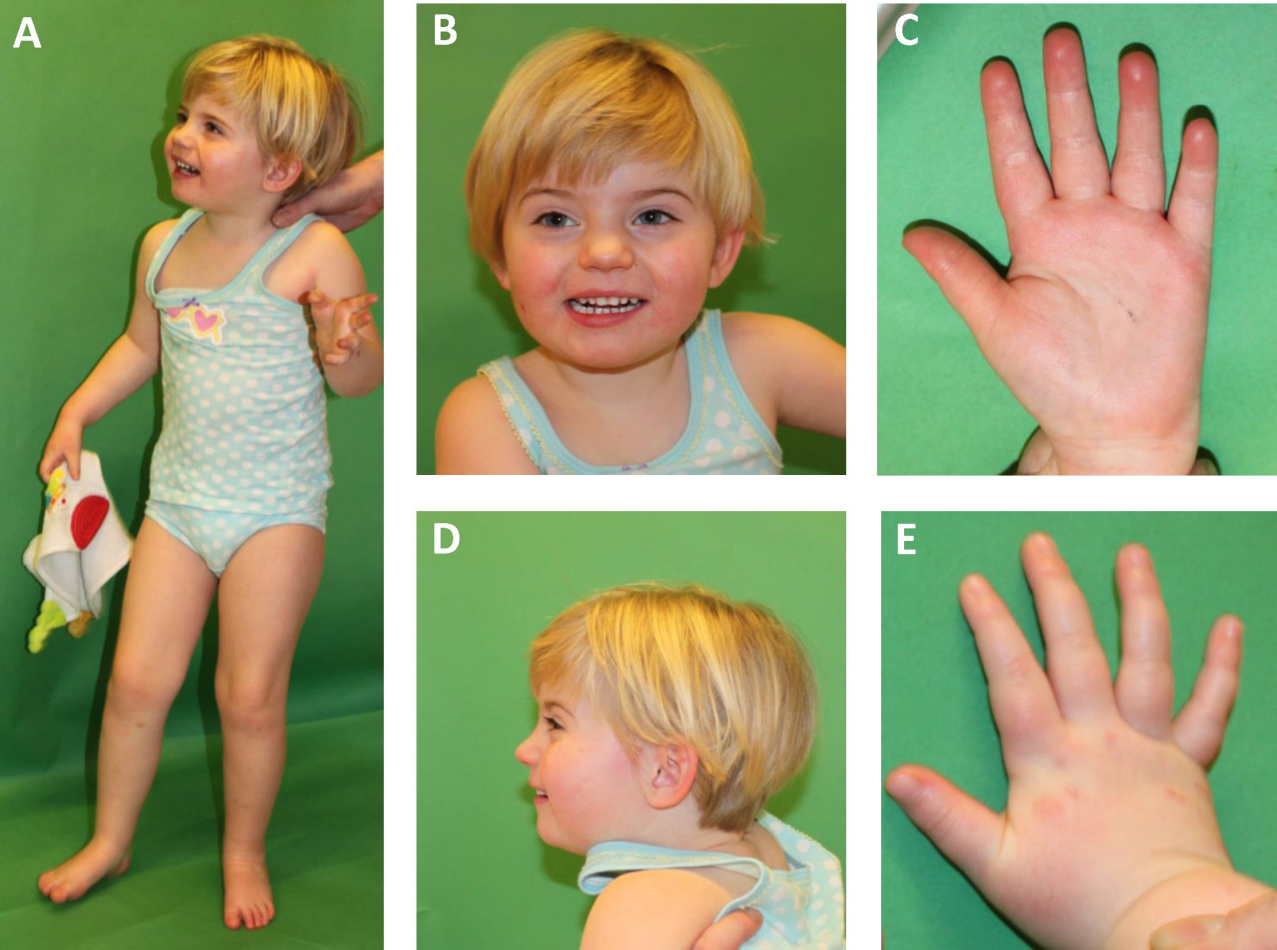
Photographs of patient #15 (*MECP2* mutation). The patient at 3 years and 6 months. The patient was diagnosed with a de novo mutation p.(Arg133Cys) in *MECP2*. Note arched eyebrows with slight synophrys, short anteverted nose, thin upper lip and smooth long philtrum.

Patient #15 was initially clinically diagnosed with a mild form of Cornelia de Lange syndrome (S1 Clinical Data, Fig 1) but carried the previously published and functionally characterized de novo mutation p.Arg133Cys in *MECP2* [10]. This mutation is the second most common mutation located within the methylated-CpG-binding domain of the MECP2 protein and is causative in 4% of all patients with MECP2-related disorders [10]. In line with previous reports, patient #15 could speak single words, did not show any regression and did not develop microcephaly [11]. She met neither main nor supportive criteria for classical or even atypical Rett syndrome. As a consequence of molecular testing the diagnosis was corrected to MECP2-associated intellectual disability. The minor facial anomalies observed in this patient appeared to be unrelated to her developmental disorder.

**Fig 2:**
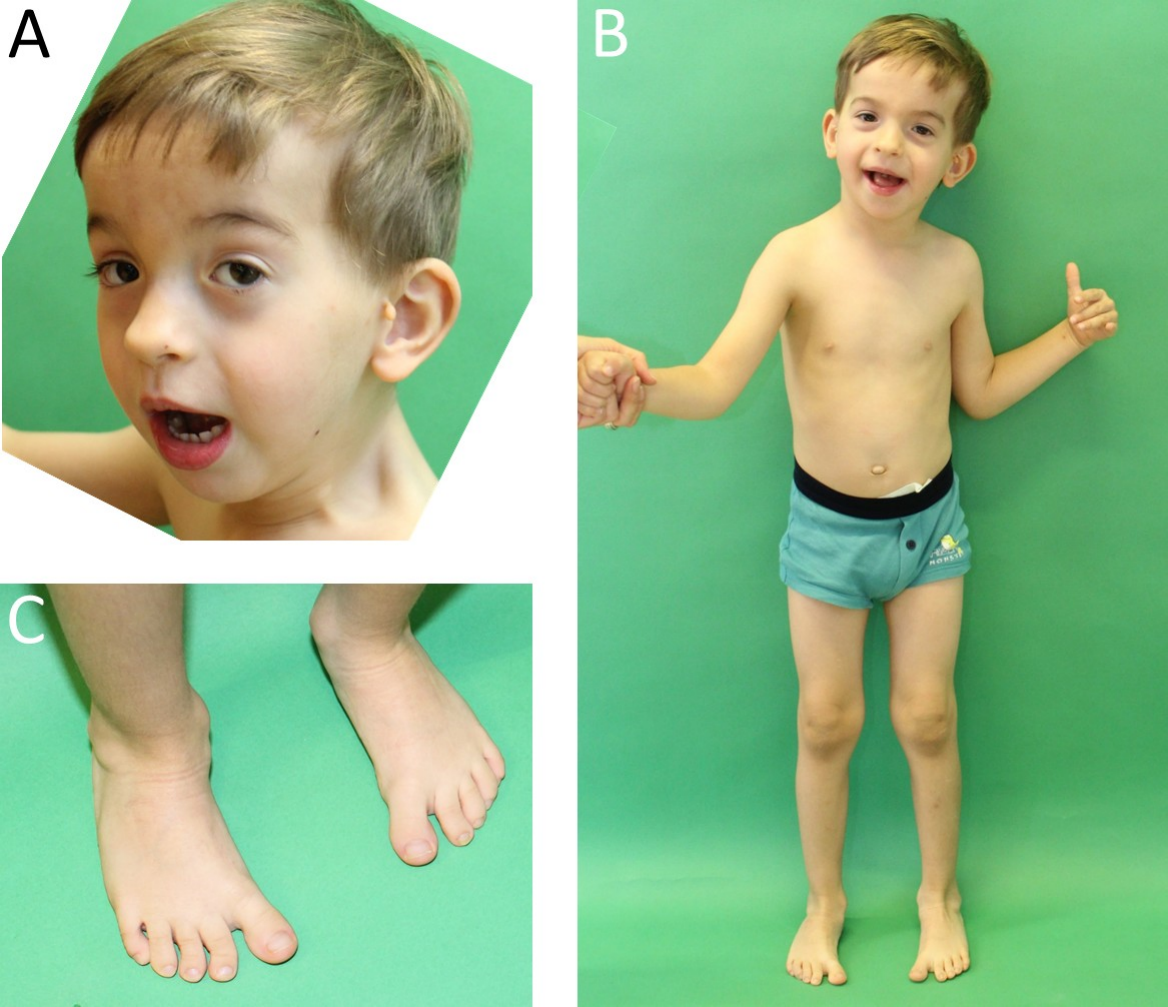
Photographs of patients #29 (*MED13L* mutation). The patient at 4 years and 2 months of age. He was diagnosed with MED13L syndrome. Note long eyelashes, broad nasal tip and open mouth appearance as well as preauricular tags.

Furthermore, we identified two patients with a MED13L related disorder that were both clinically diagnosed with Coffin-Siris syndrome (Patients #29 and #7, S1 Clinical Data, Fig 2). Notably, both patients presented with bilateral preauricular tags and showed features attributable to both MED13L and Coffin-Siris–Syndrome, such as long eyelashes and a broad nasal tip.

### Disease spectrum

The disease causing variants are summarized in S3 Table. We did not observe recurrent mutations and only two genes—*SYNGAP1* and *MED13L -* were found to be causative in two patients each. 23 patients (63.9% of the 36 solved cases, CI 47.52% - 77.58%) were diagnosed with an autosomal dominant disorder (S3 Table), 9 with an autosomal recessive disorder (25.0%, CI 13.56%– 41.26%), and 5 with X-linked syndromes (13.9%, CI 05.61% - 29.13%). Five patients, accounting for 4.7% (n = 5 / 106, CI 1.76% - 10.84%) of the whole cohort (n = 106), had potentially treatable or actively manageable medical conditions (S3 Table: patients #2, #3, #19, #23, #28). In 4 of these cases the diagnosis was not suspected clinically.

### Secondary findings and carrier status for recessive disorders

Eight secondary findings were identified in 7 of the 106 patients (6.6%, CI 3.01% - 13.23%, S4 Table). Three of these patients carried constitutional known or expected pathogenic variants in genes from the ACMG Secondary Findings minimum list *(MLH1*, *MUTYH*, *RET)* [12] and one carried compound-heterozygous VUS in *MYH7*. One further patient had a pathogenic variant in *SDHA*, associated with hereditary paraganglioma syndrome (OMIM *600857) and two patients had pathogenic and likely pathogenic mutations in *CHEK2*, which is associated with prostate and breast cancer susceptibility (OMIM +604373). We further identified *ANK2* variants (causative of long-QT syndrome, OMIM *106410), one classified as likely pathogenic and one as VUS in 1 patient each. 14.2% of patients (n = 15 / 106, CI 8.65% to 22.16%) where identified as carriers for autosomal recessive disorders (S5 Table).

### Variants of unknown significance and genes of unknown significance

VUS were determined by ACMG criteria. Eight heterozygous VUS in genes associated with autosomal dominant and X-linked disorders were identified in 7 index patients. One patient was compound heterozygous for two VUS in *COL6A3* and another patient carried biallelic VUS in *CPS21* (S6 Table). In the latter patient metabolic testing to evaluate the effect of the variants on enzyme activity could have aided variant interpretation, but he was lost during the follow-up and therefore no further classification was possible. Two VUS in the X-chromosomal genes (*MECP2* and *FLNA*) were reclassified as benign/likely benign after identification of a healthy male carrier through extensive familial testing.

Eight patients carried 13 heterozygous VUS in genes related to autosomal recessive disorders compatible with the patients’ phenotype but without any detectable changes on the second allele (S7 Table). Furthermore we identified 3 *de novo* variants and 6 compound heterozygous variants in 7 genes of unknown significance (GUS) in 7 individuals (S8 Table).

### Absence of frequent in house variants in gnomAD

**Fig 3:**
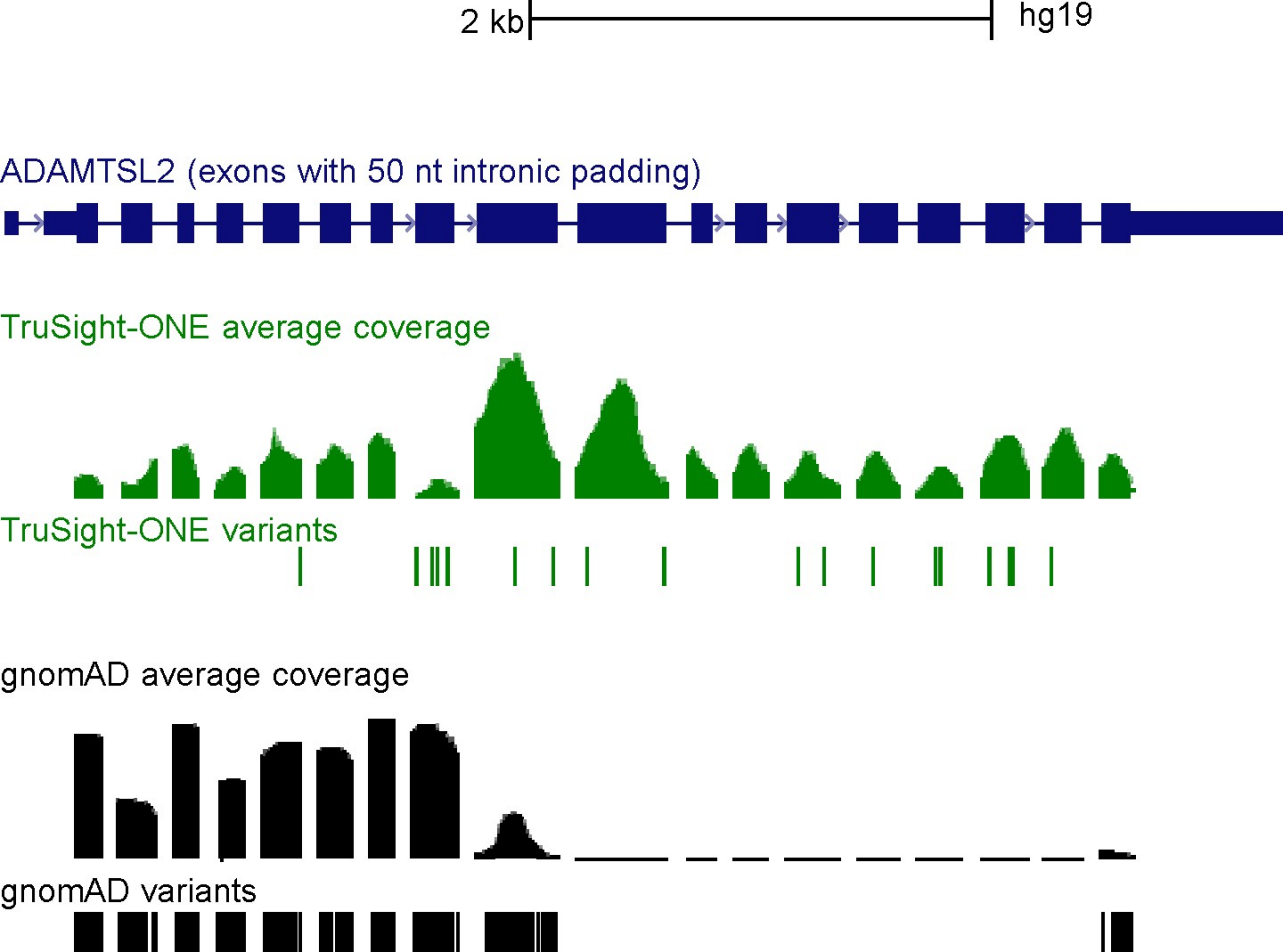
Sequence coverage and occurrence of *ADAMTSL2* variants. The data is shown in the UCSC genome browser “multi region view” (http://genome.ucsc.edu), which displays exons in full length (dark blue boxes), flanked by 50 bp of intronic sequence (dark blue vertical line). The scale on top refers to the condensed sequence shown here. The full *ADAMTSL2* gene comprises 40.6 kb of genomic DNA (chr9:136399975–136440641, hg19). Green: read coverage, target position and variants identified in this cohort; black: corresponding data in gnomAD.

The vast majority of re-occurring variants in our cohort had in-house allele frequencies (AF) similar to those in the public gnomAD database. 337 variants that were frequently found in our cases, however, are not listed in gnomAD at all. Since the absence of a variant from controls provides moderate evidence of pathogenicity (ACMG criterion PM2), we followed the ACMG’s recommendation to “confirm that the read depth in the database is sufficient for an accurate call at the variant site” [8]. As exemplarily shown for the *ADAMTSL2* gene (Fig 3), all of our in-house specific variants reside in exons which are not covered in gnomAD at all (Fig 3). Therefore, checking gnomADs target coverage and variant occurrence in additional, gnomAD-independent databases, such as an in house-database, proved essential for variant interpretation in our cohort.

## Discussion

### Diagnostic yield partial vs whole exome sequencing

During the last 3 years partial exome sequencing has increasingly been deployed for diagnostics, encompassing the whole spectrum of the Mendelian disorders. Multiple studies consistently reported overall detection rates of causative mutations of 25%-26% [3, 13, 14]. However, detection rates varied between different phenotypic categories with the highest yield (36%) attained in a group of patients with specific neurologic disorders such as seizures or ataxia [3]. Within the same study, the molecular detection rate for patients with global ID/DD, delayed speech development, autism spectrum disorder and ID/DD was reported at 24.6% [3]. A recent study based on a customized gene panel for ID/DD yielded a detection rate of 32% for mutations in known disease causing genes [15]. Earlier research projects, which specifically focused on developmental disorders, primarily included patients with severe disability, assuming to maximize the chance of finding a highly penetrant monogenic cause [16–18]. Two early studies which mainly analyzed the contribution of *de novo* mutations to sporadic severe ID varied in diagnostic yield from 16% [16] to 45–55% [18].

The Deciphering Developmental Disorders (DDD) study which, to date, is the biggest collaborative project on the genetics of developmental disorders and which is based on whole exome sequencing (WES), achieved a detection rate of causative mutations of 27% among 1 133 ID/DD patients and reported 18% of autosomal dominant, 5% of autosomal recessive and 5% of X-linked disorders [17]. A subsequent analysis of a cohort of 4 293 individuals showed that 23% of these individuals had a *de novo* truncating or (likely) pathogenic missense variant in one of the genes robustly associated with dominant ID/DD with the detection rate increased to 42% after inclusion of further candidate genes [19].

To date, the TS1 is one of the most commonly used commercially available panels and it was successfully used for genetic testing of several heterogeneous disorders such as glycogen storage disease or thoracic aortic aneurysms and dissections [20, 21] as well as for a large and unselected cohort of patients with Mendelian disorders [22]. Interestingly, the diagnostic yield attained in these studies was comparable to that of WES, varying from 26.3% in the unselected cohort [22] to 35.3% in a cohort with thoracic aortic aneurysms and dissections [21]. The TS1 based analysis of an unselected cohort of patients [22] is, to the best of our knowledge, the only available study that used the TS1 and included ID/DD cases. Since it did not, however provide sufficient information on detection rates for this specific subgroup of patients, a straight comparison to our results is not possible [22].

The TS1 contains 66 out of 94 genes known to be relevant to ID/DD and of the 28 genes missing on the panel, 8 were only recently found to be relevant to ID/DD [19]. Despite this diagnostic gap, the mutation detection rate of 34% (n = 36 / 106, CI 25.04% - 43.80%) observed in our cohort of predominantly pediatric patients with developmental disorders of various degrees was even slightly higher than the reported 27% achieved by WES [17]. We further did not attain higher detection rates in severe ID/DD as compared to mild forms, as was presumed earlier [16–18]. A previous publication from the DDD study nominated 8 most frequently mutated developmental disorders genes (*ARID1B*, *SCN1A*, *ANKRD11*, *SATB2*, *SYNGAP1*, *DYRK1A*, *MED13L* and *STXBP1*) [23]. Noteworthy, sequencing of only these genes would have solved 6 (16.7%, CI 7.49% - 32.27%) of those 36 cases we solved by TS1 sequencing. Our observation of a slightly higher diagnostic yield gained by TS1 sequencing in comparison to WES might be attributable to the relatively low number of case in our study. However, it needs to be discussed whether the naturally higher number of rare variants detected by WES, in comparison to partial exome sequencing, might impede the variant interpretation process, leading to the filtering out of potentially relevant variants, especially if variant filtering cannot be aided by parental data.

The vast majority of WES studies, both for unselected and selected cohorts, used a family or trio based approach [17, 24]. While trio sequencing is a generally recommended approach, in some cases the parents are unavailable for sequencing. For such patients, panel sequencing, which targets established disease causing genes, is considered to be a valuable approach [25].

In summary we conclude that TS1 panel sequencing provides a suitable diagnostic tool especially if WES is not covered by the insurance or if the familial trio is incomplete. However, with new genes being associated with ID/DD and with genome sequencing becoming increasingly available as an even broader diagnostic option [26], this assumption will frequently need to be reevaluated.

### Autosomal recessive ID/DD

Since a “de novo paradigm for mental retardation” [24] was published, the vast majority of studies analyzing big patient cohorts, including the recent summary of 7580 individuals, focused on heterozygous de novo mutations as the most probable disease cause [19]. However, it has been shown that autosomal recessive intellectual disability (ARID) is not rare, even in the outbred western population, and reaches an estimated frequency of 10–20% [27]. The mutation detection rate in cohorts enriched for ARID, such as consanguineous populations, even reaches 60% [28], with approximately 30% explained by ARID. We diagnosed an autosomal recessive disease in 9 patients (8.5%, n = 9 / 106, CI 4.35% - 15.54%, p = 0.1093) and thereby detected a higher proportion of patients with autosomal recessive disorders in our cohort than was described by other authors (2.2%) [15]. 8 out of these 9 cases were caused by compound-heterozygous mutations and the single homozygous mutation was identified in the only consanguineous family present in our cohort. Our findings strongly support that ARID, as a result of compound heterozygosity, is an important ID/DD cause in the outbred population and must be considered in every algorithm used for variant interpretation in diagnostic laboratories.

### Diagnostic yield by phenotype

The only phenotypic subgroup with a mutation detection rate that we noted to be lower than average in this cohort by trend, was the subgroup of patients with various types of seizures and especially patients with epileptic encephalopathy.

The reported mutation detection rate in earlier studies for this subgroup was 48% [29] for a disease-specific panel and 36% [3] for WES and was, therefore, even higher than the detection rate in broad cohorts with different forms of ID/DD. We hypothesized that such a discrepancy might be caused by the design of the TS1 panel. The direct comparison of genes included in the TS1 panel and 33 genes strongly associated with ID/DD with epilepsy (Johannes Lemke, unpublished data) showed that 9 of these genes are not included on the TS1. No data are available, however, to estimate whether mutations in these 9 genes are a frequent cause of ID/DD with epilepsy. The relatively low diagnostic yield in the subgroup of patients with epilepsy might therefore as well represent an artefact caused by a small cohort size. Based on the current data, however, this observation needs to be considered when choosing a genetic test for patients with epilepsy.

We also noted, by trend, a higher diagnostic yield for those patients with a tentative, specific clinical diagnosis (55%, n = 11 / 20, CI 34.19% - 74.19%, p = 0.1639) than the average diagnostic yield in our cohort. It seems contradicting, though, that in in this subgroup the initial clinical diagnosis could be confirmed molecularly in only 5 of 20 cases. This might be, however, due to the fact that even if the tentative diagnosis proved wrong, these patients showed specific symptoms, which led to the initial suspicion of a specific syndrome. On the basis of the tentative diagnosis these patients were clinically particularly well characterized. We assume that both the occurrence of specific symptoms as well as an in depth clinical description of the phenotype substantially aid variant interpretation.

### Unexpected molecular findings

In 6 patients the initial clinical diagnosis had to be revised according to the sequencing results (S2 Table), despite the patients having been evaluated by experienced clinical geneticists.

Patient #15, who was clinically diagnosed with a mild form of Cornelia de Lange syndrome (S1 Clinical Data, Fig 1), was identified to carry a known pathogenic *MECP2* mutation. It is interesting, in this context, that there is a putative link between the initial clinical diagnosis and *MECP2* involvement. *MECP2* encodes a chromatin regulatory protein that interacts with cohesin and cohesin-related factors, such as NIPBL. The functional link between disorders that are caused by mutations in genes encoding regulators of chromatin structure and function has been discussed in the literature [30], however a clinical overlap could so far only be observed regarding the neuronal dysfunction but not regarding facial anomalies.

We further identified two patients with MED13L related disorder that were both clinically diagnosed with Coffin-Siris syndrome (Patients #29 and #7, S1 Clinical Data, Fig 2). *MED13L* mutations were originally described as a cause of a transposition of the great arteries [31] and more recently in patients with a distinct syndromic phenotype encompassing facial anomalies, conotruncal cardial defects, and ID [32], as well as in patients with a presumably recognizable dysmorphic syndrome without cardiac involvement [33]. The common characteristic facial features were described as a broad prominent forehead, bitemporal narrowing, low set ears, upslanted palpebral fissures, flat nasal root, broad nasal tip and macrostomia with an open-mouth appearance [33]. The typical facial anomalies of Coffin-Siris syndrome include a wide mouth with thick, everted upper and lower lips, broad nasal bridge with broad nasal tip, thick eyebrows and long eyelashes [34]. Both patients from our cohort showed long eyelashes, broad nasal tip and an open mouth appearance–features partially attributed to both MED13L and Coffin-Siris syndrome. Hypertrichosis, as one of the hallmarks of Coffin-Siris syndrome, was also occasionally described in patients with MED13L syndrome [33]. Preauricular tags were rarely reported with *MED13L* mutations [32, 33], but are also listed as an associated trait of Coffin-Siris syndrome in the Possum Web database. MED13L and Coffin-Siris syndrome were never discussed as differential diagnosis to each other but our experience expands the known MED13L spectrum and suggests a previously unrecognized clinical overlap.

### Influence of non-genetic factors

Environmental factors were initially thought to account for 10–20% of patients with intellectual disability [35] but a recent study suggested that exogenic factors contribute to no more than 5% of all cases [36]. Among the environmental factors are infectious diseases, premature birth, perinatal injury (hypoxia, hypoglycemia, meningitis etc.), hypothyroidism, maternal malnutrition and fetal alcohol exposure.

Our cohort includes an illustrative example (Patient #6, S1 Clinical Data) of how an early postnatal injury can mask an otherwise recognizable genetic condition. The patient, who, on a molecular level, was diagnosed with a de novo mutation p.(Thr106Met) in *TUBB3*, underwent ventriculo-peritoneal shunting because of a hydrocephalus at the age of 8 months. A recurrent shunt infection accompanied by a ventriculitis and hemorrhage caused difficulties in postoperative brain image interpretation. Although few specific signs were visible (agenesis of the corpus callosum, severe hypoplasia and rotation of cerebellar vermis in combination with a tectal hyperplasia; S1 Fig) a tubulinopathy was not suggested as a possible differential diagnosis. The re-evaluation of the early scans after the molecular diagnosis demonstrated an MRI picture fully compatible with the tubulinopathy-spectrum [37].

### Value of variant re-evaluation

The periodic NGS data reanalysis of initially unsolved cases was shown to be very efficient, adding almost 20% of positive cases within 2 years [3]. In our cohort two additional cases could be solved by re-analysis after one year. Patient #22 carried a de novo variant in *TBL1XR1* that recently has been associated with Pierpont syndrome [38] but was classified as a gene of unknown significance at the time of the first report.

Patient #31 was compound heterozygous for two novel missense variants in *RARS2*, but presented with infantile spasms and severe diffuse supratentorial atrophy without cerebellar hypoplasia (S1 Clinical Data, S2 Fig). The variants were re-classified as causative after the observation of the missense change in two other unrelated patients with early onset epileptic encephalopathy [39] and several reports of patients with *RARS2* mutations and intact cerebellum [40, 41]. Interestingly, the reclassification of the *RARS2*-associated phenotype as an early onset mitochondrial encephalopathy [39] was further supported by elevated lactate levels in cerebrospinal fluid observed in our patient, as well as a lactate peak on her MR-spectroscopy.

This underlines how variant interpretation is not only facilitated by the rapid discovery of new disease causing genes but also by the expansion of existing phenotypes.

### Conclusions

Our data demonstrate that partial exome sequencing with Illuminas’ TS1 panel attains a diagnostic yield compatible to WES while keeping the advantages of a multiple gene panel in the diagnostic setting of ID/DD. It thereby offers an effective option for extensive genetic testing early in the diagnostic workup for children with developmental disorders. Even by studying a comparably small cohort of 106 patients we experienced several limitations of clinical diagnosis including two unrelated etiological factors in one individual, failure to recognize a known condition due to atypical presentation as well as non-identification of extremely rare syndromes with previously unknown genetic cause. We also demonstrate the significance of recessive causes for ID/DD in a non-consanguineous population and show a relatively high detection rate for immediately manageable disorders, which underlines the great impact an early molecular diagnosis can have on patient care.

## Supporting information

S1 Table Evaluation of variants regarded as causative mutations (Table 1) according to ACMG criteria. (XLSX)

S2 Table Summary of clinical information and mutation detection rates for subgroups of the cohort (including preliminary clinical diagnoses). (*) Despite the clinically suspected mosaic condition, only blood derived DNA was available for the analysis. This might be a reason for the negative test results. (**) The recently described causative gene *KMT2A* is not covered by TS1. (***) The diagnosis was confirmed by targeted microarray that revealed a deletion of exon 1 in *LIS1*.
(XLSX)

S3 Table Overview of causative mutations. (XLSX)

S4 Table Overview of secondary findings that were regarded as medically actionable. Listed are (^*a*^) (likely) pathogenic variants that are on the ACMG Secondary Findings minimum list as well as (^*b*^) (likely) pathogenic variants in genes that allowed inclusion into tailored medical surveillance programs. Patients with likely pathogenic variants or VUS in *ANK2* and *MYH7* (^*c*^) were referred for cardiologic screening.
(XLSX)

S5 Table Overview of secondary findings that were regarded as a carrier status for autosomal recessive disorders. (XLSX)

S6 Table Overview of variants of unknown significance in genes associated with the patients’ phenotype. Included are both heterozygous variants in genes related to autosomal dominant disorders or X-linked disorders as well as compound-heterozygous variants in genes related to autosomal recessive disorders. Evaluation according to ACMG criteria ranges from likely benign to likely pathogenic but all variants were considered as VUS in a clinical context. Variants that could be reclassified as (likely) benign are listed separately.
(XLSX)

S7 Table Overview of variants of unknown significance in genes causing autosomal recessive disorders that were regarded as possibly related to the patients’ phenotype but with only one variant identified in each gene in each patient. (XLSX)

S8 Table Overview of variants identified in GUS with plausible mode of inheritance. (XLSX)

S9 Table Evaluation of variants regarded as secondary findings according to ACMG criteria. (XLSX)

S1 Clinical Data Supplementary clinical information. The supplementary clinical information includes data from patients listed in the accompanying tables. Patients are identified by a patient-ID listed with the Information.
(DOCX)

S1 Fig Brain MRI of patient #6 (*TUBB3* mutation). The patient carries a missense mutation in TUBB3. T1 (A) and T2-weighted images at 6 days (B-C) and 5 months (D-F) showing hypoplastic and mildly everted cerebellar vermis (white arrow in A, black arrows in D and F). Also note dilatation of the 4th ventricle and enlarged posterior fossa (marked with white and black asterisks in (A) and (D) respectively), in combination with hyperplastic tectum (arrowheads A and D), as well as hypoplastic brain stem and absent corpus callosum. MRI scans further show progressive enlargement of the lateral ventricles (single asterisk in B and double asterisks in E) and dysplastic basal ganglia (asterisks in C).
(JPG)

S2 Fig Brain MRI of patient #31 (*RARS2* mutations). The patient was diagnosed with compound heterozygous mutations in *RARS2*. (A-C) T2 weighted MRI scans at the age of 23 days demonstrating normal brain morphology. (D-F) MRI scans at 22 months of age showing mild atrophy of the cerebellar vermis (black arrow head), supratentorial atrophy with enlarged extraaxial space (asterisks) and wide sulci. (G-I) Normal control images.
(JPG)
