# Correction: Diagnostic value of partial exome sequencing in developmental disorders

**DOI:** 10.1371/journal.pone.0239959

**Published:** 2020-09-24

**Authors:** Laura Gieldon, Luisa Mackenroth, Anne-Karin Kahlert, Johannes R. Lemke, Joseph Porrmann, Jens Schallner, Maja von der Hagen, Susanne Markus, Sabine Weidensee, Barbara Novotna, Charlotte Soerensen, Barbara Klink, Johannes Wagner, Andreas Tzschach, Arne Jahn, Franziska Kuhlee, Karl Hackmann, Evelin Schrock, Nataliya Di Donato, Andreas Rump

## Notice of republication

An incorrect version of Fig 2 was published in error. This article was republished on September 4, 2020, to correct for this error. Please download this article again to view the correct version.
