# Supplementary material for: Diagnostic value of partial exome sequencing in developmental disorders
Source: PLoS One. 2018 Aug 9;13(8):e0201041. doi: 10.1371/journal.pone.0201041 (PMC6084857; doi:10.1371/journal.pone.0201041)
Supplement: S1 Clinical Data — The supplementary clinical information includes data from patients listed in the accompanying tables. Patients are identified by a patient-ID listed with the Information. (DOCX) [file pone.0201041.s010.docx]

**Supplementary clinical data**

**Patient #24**

**SYNGAP1 (OMIM** # **612621, autosomal dominant)**

**NM_006772.2:c.763-1G>A, het, de novo**

The female patient was 12 years old at first presentation. She was born after an uneventful pregnancy to non-consanguine Syrian parents. She started walking at 2 years and only spoke few words at the time of presentation. Facial minor anomalies included bilateral epicanthus, a thin upper lip vermilion and an everted lower lip. Body measurements were normal. The patient showed moderate to severe intellectual disability and received special schooling. A brain MRI at the age of 5 years was unremarkable.

**Patient #8**

**SYNGAP1 (OMIM** # **612621, autosomal dominant)**

**NM_006772.2:c.3826dup, p.Asp1276Glyfs*7,het, de novo**

A 3 years and 6 months old girl was born at term (39. gestational week (GW)) after an uneventful pregnancy. Her birth measurement including occipitofrontal head circumference (OFC)of 34 cm (- 0,46SD) were normal. At the age of 3 months she was diagnosed with microcephaly and developmental delay. At the last follow-up she could only stand with support and showed no speech development. Her length was 92 cm (- 1,86 SD), weight 13,2 kg (- 1,14 SD) and head circumference 46 cm (-3,38 SD). She showed an everted lower lip and relatively large ears. Brain MRI at the age of 2 years was normal. She showed no additional neurological symptoms.

**Patient #7**

**MED13L (OMIM** # **616789, autosomal dominant)**

**NM_015335.4:c.5849T>C, p.(Leu1950Pro), het, de novo**

This girl was born in the 40^th^ GW to healthy German non-consanguineous parents. She showed normal body measurements but was referred to the genetic counselling at 1 month of age due to a heart defect (pulmonary stenosis with patent foramen ovale, no operation needed), feeding difficulties, pes adductus and bilateral skin tags. Her motor and speech development was delayed. She walked independently at the age of 2.5 years and spoke first word at 3 years of age. By the age of 7 years she developed corneal irregularity. She had autistic features, autoagressive behavior and focal epilepsy (first episode at the age of 4 years).

At the last follow-up at 7 years and 1 month her height was 120.5 cm (- 0,67 SD), weight 28 kg (0,88 SD) and head circumference was 53 cm (1,12 SD). She presented with downslanted palpebral fissures, long eyelashes, broad nasal tip, broad lips, open mouth appearance, preauricular skin tags, hairy arms, legs and spine, inverted nipples, dysplastic toe nails (especially of the fifth toe), as well as a unilateral sandal gap. The girl showed a considerable face coarsening over the years **(Supplementary Figure 2, A-G).** A brain MRI-scan showed slightly enlarged extra axial spaces but was unremarkable otherwise. Due to the combination of facial minor anomalies with dysplastic nails Coffin-Siris syndrome was discussed as a tentative diagnosis prior to panel sequencing.

**Patient #29**

**MED13L (OMIM** # **616789, autosomal dominant)**

**NM_015335.4:c.3276delinsAA, p.(Asn1093Lysfs*32), het, de novo**

The patient was born to healthy German non-consanguineous parents after an uneventful pregnancy in the 39^th^ GW with normal body measurements. He was examined at the age of 4 years due to moderate developmental delay. He spoke first words at 3 years and walked without support at 3 years and 3 months. At 4 years his height was 100 cm (- 0,99 SD), his weight was 14.7kg (- 1,08 SD) and his head circumference was 49 cm (- 1,73 SD). He presented with a high and broad forehead, down-slanted palpebral fissures, mildly arched eyebrows, long eyelashes, a broad nasal tip, an open mouth appearance, low set ears, bilateral preauricular skin tags and bilateral sandal gaps **(Supplementary Figure 2, H-J).** A brain MRI showed normal morphology. The patient did not have any major anomalies. A tentative diagnosis of Coffin-Siris syndrome was discussed prior to genetic testing.

**Patient #31**

**RARS2 (OMIM** # **611523, autosomal recessive)**

**NM_020320.4: c.1544A>G, p.(Asp515Gly, het, pat/**

**NM_020320.4: c.16C>T, p.(Arg6Cys), het, mat**

The girl was born to non-consanguineous parents after an uneventful pregnancy in the 42^nd^ GW with normal body measurements. From the 3^rd^ day of life she developed seizures (8-10 attacks per day) that were therapy-resistant. She was fed through a PEG feeding tube placed at the age of 9 months.

At the last follow-up at 1 year and 5 months she had no developmental progress (no visual fixation, no head control). Her length and weight were normal (length 82 cm, 0,46 SD and weight 10,5 kg, -0,08 SD), but she was severely microcephalic (head circumference 42,5 cm, -4,16 SD). The patient did not show any minor anomalies and had no major malformation. CFS analysis showed elevated levels of lactate (2.79 mmol/l, reference: 1.20-2.19 mmol/l). The brain MRI was unremarkable at the first week of life but showed pronounced diffuse atrophy both of the cortex and supratentorial white matter at the follow-up examinations at the age of 1 year and 2 months **(Supplementary Figure 3)**. MR-spectroscopy showed locally elevated lipid concentrations as well as slightly elevated lactate concentrations of the basal ganglia.

**Patient #6**

**TUBB3 (OMIM** # **614039, autosomal dominant)**

**NM_001197181.1: c.317C>T, p.(Thr106Met), het, de novo**

This girl was born in the 41^st^ gestational week. Intrauterine growth retardation was initially observed in prenatal examinations and birth measurements were: length 47cm (-2,36 SD), weight 2765g (-1,9 SD), OFC 32cm (-2,46 SD)). A postnatal MRI scan revealed a complex brain malformation with agenesis of the corpus callosum, vermis hypoplasia, hypoplasia of the brain stem and hydrocephalus internus. A shunt operation was performed at 8 months but hemorrhage and ventriculitis occurred as complications, making a revision operation necessary. No significant cognitive or motor development was ever observed. At 4 years of age head control was possible for app. 10 minutes a time. In addition multifocal epilepsy with complex focal seizures was diagnosed and proved refractory to treatment. Body measurements at 4 years showed progressive brachy-microcephaly (OCF 40,5cm, -7,46 SD) and consistent short stature (length 93,5cm, -2,55 SD). Facial features such as strabismus and unilateral ptosis were attributed to the shunt-operation.

**Patient #15**

**MECP2 (OMIM** # **312750, x-linked dominant)**

**NM_004992.3: c.397C>T, p.(Arg133Cys), het, de novo**

The girl was born via cesarean section in the 39^th^ GW. Body measurements were normal (length 51 cm, 45th centile; weight 3690 g, 77th centile, OFC 37cm, 96th centile). The patient was able to sit at 6-7 months and walked unsupportedly at 18 months. She spoke her first words at 12 months and learned approximately 10 words, with no further speech development. She showed no developmental stagnation. At the last follow-up at 3 years and 6 months her developmental age was comparable to that of a 16-18 months old child. Body measurements were normal (length 99.5 cm, 41th centile; weight 16.9 kg, 71th centile; OFC 49.2 cm, 31th centile). A brain MRI was normal except for slightly enlarged extra-axial spaces. Facial features included arched eyebrows with slight synophrys, short anteverted nose, thin upper lip and smooth long philtrum **(Supplementary figure 1).** The patient also had slightly tapering fingers. Facial gestalt was suggestive for mild Cornelia-de-Lange syndrome (CdLs).
