# Supplementary figures and images for: Diagnostic value of partial exome sequencing in developmental disorders
Source: PLoS One. 2018 Aug 9;13(8):e0201041. doi: 10.1371/journal.pone.0201041 (PMC6084857; doi:10.1371/journal.pone.0201041)

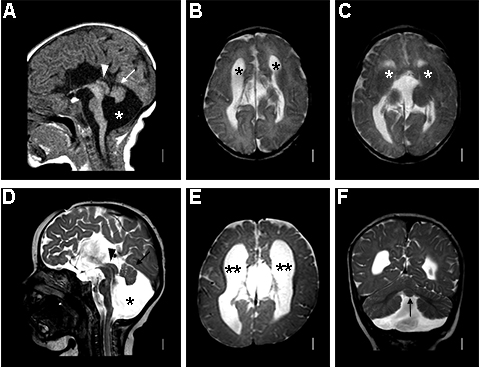

Supplement: S1 Fig — The patient carries a missense mutation in TUBB3. T1 (A) and T2-weighted images at 6 days (B-C) and 5 months (D-F) showing hypoplastic and mildly everted cerebellar vermis (white arrow in A, black arrows in D and F). Also note dilatation of the 4th ventricle and enlarged posterior fossa (marked with white and black asterisks in (A) and (D) respectively), in combination with hyperplastic tectum (arrowheads A and D), as well as hypoplastic brain stem and absent corpus callosum. MRI scans further show progressive enlargement of the lateral ventricles (single asterisk in B and double asterisks in E) and dysplastic basal ganglia (asterisks in C). (JPG) [file pone.0201041.s011.jpg]

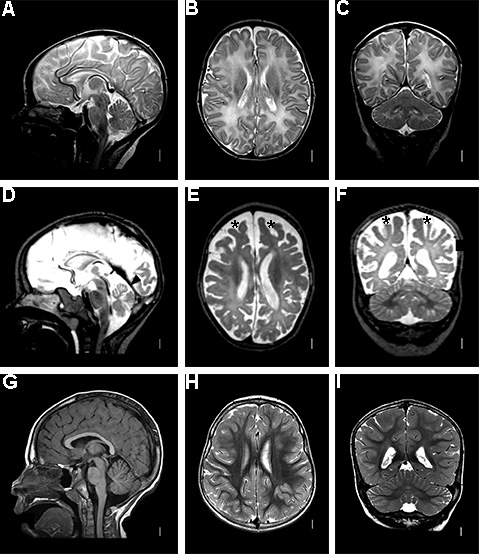

Supplement: S2 Fig — The patient was diagnosed with compound heterozygous mutations in RARS2. (A-C) T2 weighted MRI scans at the age of 23 days demonstrating normal brain morphology. (D-F) MRI scans at 22 months of age showing mild atrophy of the cerebellar vermis (black arrow head), supratentorial atrophy with enlarged extraaxial space (asterisks) and wide sulci. (G-I) Normal control images. (JPG) [file pone.0201041.s012.jpg]
